# Sustained disease control in ovarian carcinosarcoma treated with postoperative hyperthermic intraperitoneal chemotherapy and targeted maintenance: a case report

**DOI:** 10.3389/fmed.2026.1853847

**Published:** 2026-06-10

**Authors:** Shuo Xu, Xinjun Li, Xiangnan Zhang, Ren Xu, Lu Sang, Yanan Ren, Ning Sun, Xiao Shao, Shengpu Wang

**Affiliations:** 1 Department of Gynecology, Hebei General Hospital, Shijiazhuang, China; 2 Department of Pathology, Hebei General Hospital, Shijiazhuang, China; 3 Department of Obstetrics, The Second Hospital of Hebei Medical University, Shijiazhuang, China

**Keywords:** case report, disease control, hyperthermic intraperitoneal chemotherapy, ovarian carcinosarcoma, targeted maintenance

## Abstract

**Objective:**

Ovarian carcinosarcoma (OCS) is a rare and highly aggressive malignancy with no established standard treatment and a poor prognosis. This case describes sustained disease control achieved through the combination of cytoreductive surgery followed by postoperative hyperthermic intraperitoneal chemotherapy (postoperative HIPEC) and molecularly guided sequential maintenance therapy.

**Methods:**

We report the clinicopathological features, diagnosis, and treatment of a 60-year-old woman with advanced OCS. After cytoreductive surgery, she received postoperative HIPEC followed by adjuvant chemotherapy nab-paclitaxel and carboplatin, then sequential maintenance therapy with bevacizumab followed by olaparib based on homologous recombination deficiency (HRD) positivity. Relevant literature on OCS management was reviewed.

**Results:**

The patient remained progression-free with good performance status at 24 months of follow-up.

**Conclusion:**

This hypothesis-generating case suggests that for selected patients with OCS, a multimodal approach incorporating aggressive cytoreductive surgery, postoperative HIPEC, and HRD-guided sequential maintenance therapy may warrant further investigation.

## Introduction

Ovarian carcinosarcoma (OCS), also known as malignant mixed Müllerian tumor, accounts for 1–4% of all ovarian tumors and is significantly more aggressive than other ovarian cancer histotypes (1–3). OCS is a biphasic tumor comprising malignant epithelial (carcinomatous) and stromal (sarcomatous) components, with various subtypes including serous and endometrioid. Approximately 90% of OCS cases present with extra-ovarian spread (4, 5).

Optimal cytoreductive surgery (CRS) followed by platinum-based chemotherapy remains standard of care. However, the prognosis is poor, as most patients experience rapid recurrence and show limited response to subsequent therapies (6, 7).

Emerging molecular insights indicate that homologous recombination deficiency (HRD) and BRCA mutations may represent therapeutic targets in OCS (8–13). Although data on poly(ADP-ribose) polymerase inhibitors (PARPis) in OCS remain limited, several case series have reported clinically meaningful benefits (14, 15).

To address the high risk of peritoneal recurrence, we incorporated postoperative hyperthermic intraperitoneal chemotherapy into the initial treatment regimen. Here, we report a case of advanced OCS managed with a multimodal approach combining CRS and HIPEC, followed by HRD-guided sequential maintenance therapy with bevacizumab then olaparib, which resulted in sustained disease control. We hypothesize that this strategy warrants further investigation in this challenging disease.

## Case presentation

### Clinical data

A 60-year-old postmenopausal woman presented with a 2-month history of constipation, without vaginal bleeding, discharge, or abdominal pain, for which she initially did not seek medical attention. One week before admission, her constipation worsened, accompanied by abdominal distension and difficulty eating. A pelvic mass was initially identified at a local hospital, but no paracentesis, biopsy, or other diagnostic procedure was performed there. She was admitted to our hospital on January 9, 2024, for further evaluation. On admission, her vital signs were within normal limits: blood pressure 125/78 mmHg, heart rate 78 bpm, respiratory rate 16/min, and afebrile (36.8 °C). Abdominal examination revealed distension with positive shifting dullness, but no tenderness, rebound tenderness, or guarding. A large, firm, non-mobile pelvic mass was palpable on bimanual pelvic examination, extending to the left adnexal region. No inguinal, supraclavicular, or axillary lymphadenopathy was detected. ECOG performance status at presentation was 2. The patient had a documented history of hypertension grade 3 (high-risk) managed with amlodipine 5 mg daily but no prior history of diabetes or malignancy. She had no previous abdominal or pelvic surgeries. No family history of breast, ovarian, endometrial, or other hereditary cancers was reported. Genetic counseling was provided preoperatively, and germline BRCA testing was offered and completed postoperatively. No significant psychosocial barriers to treatment adherence or follow-up were identified.

Transvaginal ultrasound revealed a 158 mm × 126 mm × 84 mm mixed echogenicity mass behind the uterus with solid and cystic components, as well as free fluid in the abdominopelvic cavity. Contrast-enhanced computed tomography showed a 104 mm × 110 mm × 90 mm heterogeneous pelvic mass with unclear borders involving the sigmoid colon, rectum, and uterus, along with omental and peritoneal nodules (Figure 1). PET-CT confirmed a hypermetabolic pelvic mass, peritoneal metastases, and malignant ascites. Colonoscopy revealed extrinsic compression of the rectosigmoid colon.

**Figure 1:**
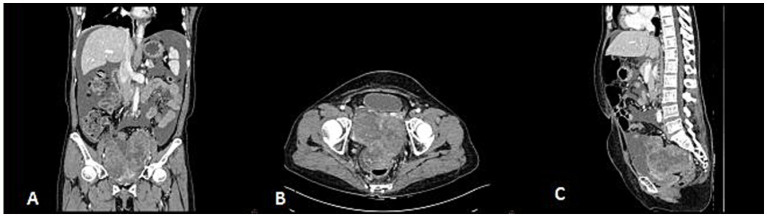
Contrast-enhanced computed tomography (CT) images of the pelvic mass. **(A)** Coronal, **(B)** axial, and **(C)** sagittal views showing a heterogeneous pelvic mass.

The initial diagnosis was ovarian malignancy. With a Suidan score <3, the patient underwent exploratory laparotomy on January 18, 2024.

### Intraoperative findings

Approximately 2,500 mL of serosanguineous ascites was present in the pelvic and abdominal cavity; 1,000 mL was retained for analysis. An irregular tumor measuring approximately 20 cm × 15 cm × 15 cm originated from the left ovary, with multiple cystic changes and a grayish-brown color. It adhered densely to the surrounding intestines, bilateral pelvic walls, and the posterior uterine wall, resulting in a frozen pelvis. The right ovary was approximately 8 cm and mixed cystic-solid. Both ovaries extensively invaded the anterior rectal wall.

Frozen section of the right adnexal mass indicated well-differentiated serous carcinoma. Widespread carcinomatosis was confirmed, with tumor nodules on the diaphragmatic surfaces, mesentery, peritoneum, appendix, and omentum. The largest omental deposit measured 6 cm. A 3 cm hard nodule was found in the omentum near the splenic inferior pole. No tumor nodules were seen on the bladder, liver, stomach, or spleen. Enlarged para-aortic lymph nodes were palpated.

Due to rectal invasion, a rectal resection with low intestinal anastomosis was performed to preserve anal function.

### Surgical procedure

The patient underwent abdominal modified radical hysterectomy, bilateral salpingo-oophorectomy, appendectomy, omentectomy, para-aortic lymphadenectomy, resection of abdominopelvic tumor deposits, and partial proctectomy. Complete cytoreduction with no gross residual disease (R0) was achieved. Specifically, all visible peritoneal deposits were resected, including nodules on the diaphragmatic surfaces, mesentery, peritoneum, appendix, and omentum. The omental nodule near the splenic inferior pole was completely excised. The rectosigmoid colon was resected en bloc with the pelvic tumor, followed by low intestinal anastomosis. Systematic inspection of the bilateral paracolic gutters, liver surface, stomach, and spleen confirmed no residual macroscopic disease. Estimated blood loss was 1,000 mL, requiring transfusion of 6 units of leukocyte-depleted red blood cells and 600 mL of fresh frozen plasma, which was administered without incident.

### Pathological findings

Postoperative pathology confirmed ovarian carcinosarcoma (malignant mixed Müllerian tumor) in both ovaries, with components of high-grade serous carcinoma, undifferentiated carcinoma, squamous cell carcinoma, and high-grade sarcoma (Figure 2). Immunohistochemistry showed mutant-type p53, focal ER/PR positivity, Ki-67 ~ 80%, and intact mismatch repair proteins (pMMR). HRD status and BRCA1/2 mutations were assessed using formalin-fixed paraffin-embedded tumor tissue via next-generation sequencing (AmoyDx HRD Focus Panel, Amoy Diagnostics, China). The HRD score was 54, with a positive threshold defined as ≥42 according to the manufacturer’s validation. No pathogenic or likely pathogenic BRCA1/2 mutations were identified in either germline (peripheral blood) or somatic (tumor tissue) testing. No other actionable alterations, including RAD51C, RAD51D, PALB2, or TP53, were detected beyond a known TP53 mutation. Lymph node metastasis was present in 2 of 6 nodes. Based on the presence of a 6 cm omental deposit, the final FIGO stage was revised to IIIC.

**Figure 2:**
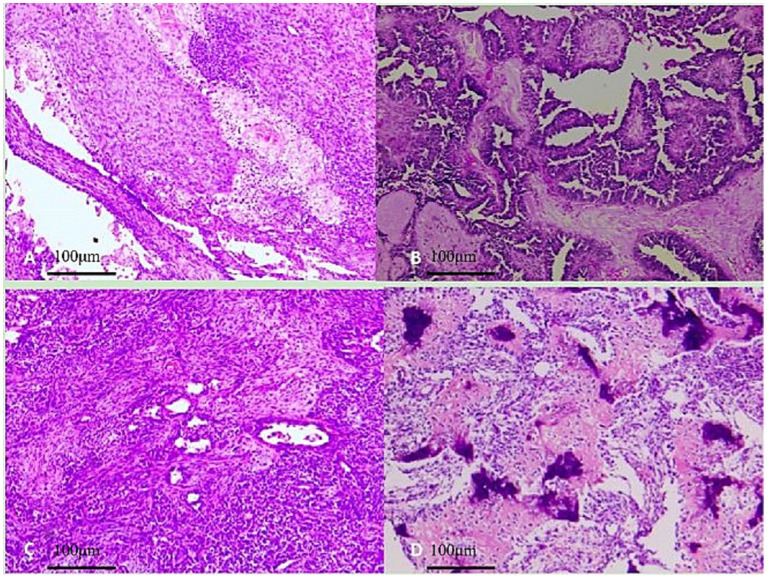
Representative histopathological images of ovarian carcinosarcoma (hematoxylin and eosin stain; original magnification ×400; scale bar = 100 μm). **(A)** Squamous cell carcinoma component with keratinization; **(B)** High-grade serous carcinoma component with psammoma bodies; **(C)** Undifferentiated carcinoma component with high nuclear pleomorphism; **(D)** High-grade sarcoma component with spindle cell morphology and brisk mitotic activity.

### Diagnostic assessment

The differential diagnosis included high-grade serous carcinoma (HGSC), primary peritoneal carcinoma, metastatic gastrointestinal malignancy, and other Müllerian tumors. HGSC was considered due to the pelvic mass and markedly elevated CA125; however, the presence of both carcinomatous and sarcomatous components on final histopathology confirmed ovarian carcinosarcoma. Primary peritoneal carcinoma was excluded by the absence of diffuse peritoneal involvement without a dominant ovarian mass. Colonoscopy revealed only extrinsic compression without mucosal lesions, ruling out primary colorectal cancer. The diagnosis of OCS was definitively established by postoperative pathology demonstrating biphasic histology (malignant epithelial and stromal components), a mutant-type p53 pattern, intact mismatch repair proteins (pMMR), and sarcomatous differentiation. As noted by Palisciano et al., benign adnexal masses may occasionally mimic malignancy on multimodal imaging (16); however, in this case, intraoperative findings and histopathology unequivocally confirmed malignancy.

### Treatment and follow-up

Complete cytoreduction with no gross residual disease (R0) was achieved. Despite complete CRS, most patients with ovarian cancer recur in the peritoneal cavity. Postoperative hyperthermic intraperitoneal chemotherapy targets residual microscopic disease by delivering heated chemotherapy directly to this site. Given that ovarian cancer often spreads peritoneally, we used postoperative HIPEC as a locoregional treatment to enhance chemotherapy efficacy beyond systemic therapy alone. The chemotherapeutic agent was selected by consensus among gynecologic, surgical, and medical oncologists based on histology and prior treatment response.

Postoperative HIPEC procedure: The patient received cisplatin perfusion (50 mg/m^2^) on three consecutive days within 1 week after surgery (closed technique, 41–43 °C, 90 min per session). This regimen follows our institutional protocol for extensive peritoneal disease and differs from the single intraoperative HIPEC procedure evaluated in randomized trials for epithelial ovarian cancer. Intravenous hydration was administered for renal protection and serum creatinine was monitored daily. Adjuvant chemotherapy consisted of six cycles of nab-paclitaxel (390 mg) and carboplatin (590 mg) intravenously every 21 days. Nab-paclitaxel was chosen over conventional paclitaxel due to its improved tumor penetration, reduced need for corticosteroid premedication, and favorable tolerability profile in older patients with aggressive histologies such as ovarian carcinosarcoma, consistent with our institutional practice. Bevacizumab (800 mg) was added intravenously every 21 days starting from the second cycle of chemotherapy and continued for 22 cycles.

After completion of bevacizumab and adjuvant chemotherapy, maintenance olaparib (300 mg twice daily) was initiated sequentially, provided that hematologic recovery (absolute neutrophil count ≥1.5 × 10^9^/L, platelets ≥100 × 10^9^/L) and normal renal/liver function were achieved. Thus, bevacizumab and olaparib were administered sequentially, not concomitantly. Grade 1 fatigue and grade 1 anemia occurred during olaparib maintenance, which were managed conservatively without dose reduction or treatment interruption. No grade ≥3 adverse events were observed. Maintenance therapy is planned to continue for up to 24 months in the absence of progression or unacceptable toxicity.

Preoperative CA125 was 1,231 U/mL. After surgery and three cycles of adjuvant chemotherapy, CA125 decreased to 22 U/mL, and at the most recent follow-up, it remained within normal limits (<35 U/mL). ECOG performance status improved from 2 at presentation to 0 after completion of adjuvant therapy. Follow-up contrast-enhanced CT performed at 6, 12, 18, and 24 months post-surgery showed no evidence of disease recurrence or new peritoneal implants. A timeline of the diagnosis and treatment pathway is presented in Figure 3.

**Figure 3:**
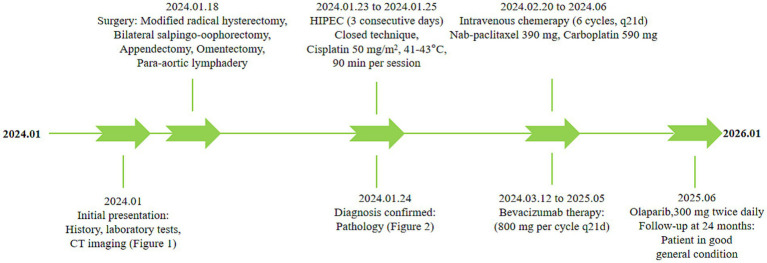
A timeline figure summarizing the case diagnosis and treatment pathway.

The patient reported gradual resolution of constipation and abdominal distension after surgery and chemotherapy. She described fatigue as the most burdensome symptom during olaparib maintenance but rated her overall quality of life as “good” (EQ-5D-5L visual analog scale: 75/100 at 24 months). She expressed satisfaction with the treatment course and willingness to continue follow-up. No significant financial or psychosocial barriers to adherence were identified.

## Discussion

Ovarian carcinosarcoma is an extremely rare and highly aggressive ovarian malignancy characterized by strong invasiveness, rapid proliferation, and early metastasis. Median overall survival remains less than 2 years (17). Although optimal cytoreductive surgery with platinum-based chemotherapy is the current standard, outcomes are poor due to rapid recurrence and limited chemotherapy sensitivity (18).

In an effort to mitigate the high risk of peritoneal recurrence, we added postoperative hyperthermic intraperitoneal chemotherapy. Postoperative HIPEC provides mechanical washout, direct hyperthermic cytotoxicity, and enhanced chemoperfusion, and has been shown to improve survival in ovarian cancer (19). In OCS, CRS with HIPEC has been associated with favorable abdominal disease control and a low rate of malignant obstruction (20, 21). Guerra et al. (22) considered hyperthermic intraperitoneal chemotherapy (HIPEC), which delivers heated chemotherapy directly into the peritoneal cavity, enhancing local cytotoxicity and offering a potential therapeutic strategy. In ovarian cancer, HIPEC has been shown to significantly improve OS (22). Given the lower response rate of peritoneal disease to systemic therapy and its potential to cause debilitating symptoms, the addition of HIPEC during cytoreduction appears to be a feasible option for achieving peritoneal disease control in OCS patients. Collaborative studies with larger cohorts and longer follow-up may further clarify the role of CRS with HIPEC in OCS (23).

In this case, postoperative HIPEC served as a regional intensive treatment to reduce peritoneal tumor burden — the primary site of OCS dissemination. However, it is important to note that the observed 24-month progression-free survival cannot be causally attributed to any single intervention, including HIPEC, olaparib, or their potential interaction. The favorable outcome should be viewed as an observational finding from a single case, not as evidence of treatment efficacy or synergy.

Emerging molecular data suggest that a subset of OCS tumors harbor HRD or BRCA mutations, rendering them potentially susceptible to PARP inhibitors (14). In this case, next-generation sequencing identified HRD positivity, which provided a clear rationale for subsequent precision maintenance therapy. The mechanism of action of PARP inhibitors is synthetic lethality, particularly in HRD-positive tumor cells. In addition to olaparib, other PARP inhibitors such as niraparib have also shown potential activity in ovarian carcinosarcoma (23). Future studies should compare the efficacy and safety profiles of different PARP inhibitors, either as monotherapy or in combination with anti-angiogenic agents, in molecularly selected OCS patients.

Liang et al. (7) conducted a cohort study of 51 OCS patients and found that complete cytoreduction with no residual disease was the only independent prognostic factor. They recommended whole-exome sequencing, especially HRD testing, to assess prognosis and guide targeted therapy (7). Dinicu et al. (14) and St Laurent et al. (24) separately reported case series of OCS patients treated with PARP inhibitors, observing clinically meaningful benefits in both frontline and recurrent settings. Research by Ji et al. (9) suggested that age at diagnosis, tumor diameter, Ki-67 index, and ascites volume may be prognostic factors for OCS. Patients with HRD positivity or BRCA mutations might benefit from PARPis. Sonoda et al. (25) provided evidence that hereditary ovarian carcinosarcoma may be caused by BRCA2 mutations, with both histological components originating from the same progenitor cell. Bevacizumab was the first targeted therapy approved for newly diagnosed and recurrent advanced ovarian cancer, and it is associated with improved progression-free survival regardless of clinical risk (26).

We hypothesize, as a purely speculative and unproven biological hypothesis, that extensive DNA damage induced by HIPEC might create a state of replicative stress in HRD-positive tumor cells, potentially increasing their vulnerability to subsequent PARP inhibition through synthetic lethality. This hypothesis is not substantiated by direct evidence from this case and requires dedicated preclinical and clinical investigation. Additionally, the sequential use of bevacizumab followed by olaparib may have contributed to disease control through complementary mechanisms, but this remains speculative.

### Limitations and future prospects

As a single case report, this study has inherent limitations. While the success of an individual case can generate hypotheses, it cannot establish universal clinical guidelines. Several additional limitations should be acknowledged. First, the favorable outcome cannot be attributed to any single intervention, as the patient received CRS, postoperative HIPEC, chemotherapy, bevacizumab, and olaparib concurrently (attribution bias). Second, the 24-month follow-up, while encouraging, is insufficient to claim long-term disease control. Third, the generalizability of this strategy to other HRD-positive/BRCA-wildtype OCS patients remains uncertain. Fourth, the hypothesis regarding postoperative HIPEC-induced DNA damage sensitizing to PARP inhibition is speculative. Finally, publication bias may favor positive outcomes; negative or null results from similar cases are less likely to be published. The efficacy, safety, optimal drug combinations, and perfusion timing of postoperative HIPEC combined with targeted therapy for OCS require confirmation through large-scale, multicenter prospective clinical trials. Furthermore, deeper molecular subtyping of OCS and exploration of therapeutic targets beyond BRCA will be essential for achieving more precise, personalized treatment in the future.

## Conclusion

In summary, this hypothesis-generating case report suggests that for ovarian carcinosarcoma — traditionally considered to have an extremely poor prognosis — a multimodal approach involving aggressive cytoreductive surgery combined with postoperative HIPEC followed by molecularly guided sequential maintenance therapy may be a promising personalized treatment strategy. While this case demonstrates sustained 24-month progression-free survival in a single patient with advanced OCS (FIGO stage IIIC), it remains hypothesis-generating rather than definitive evidence of treatment efficacy. This hypothesis-generating observation warrants further investigation in larger and more robust clinical studies. Future evidence-based data are needed to refine and promote this therapeutic paradigm.

## Data Availability

The original contributions presented in the study are included in the article/supplementary material, further inquiries can be directed to the corresponding author.
